# Genomic analysis of methicillin-resistant *Staphylococcus aureus* strain SO-1977 from Sudan

**DOI:** 10.1186/s12866-019-1470-2

**Published:** 2019-06-11

**Authors:** Mohamed S. Ali, Nurulfiza M. Isa, Faisal M. Abedelrhman, Tahani B. Alyas, Sara E. Mohammed, Abdallah E. Ahmed, Zainab S. A. Ahmed, Nyok-Sean Lau, Mohamed I. Garbi, Abdullah Al-Ashraf Amirul, Almeen O. Seed, Rihab A. Omer, Sofia B. Mohamed

**Affiliations:** 1 Department of Bioinformatics and Biostatistics, National University Research Institute- National University, Khartoum, Sudan; 2 Department of Cell and Molecular Biology, Faculty of Biotechnology and Bimolecular Sciences, Universiti Putra Malaysia, Selangor, Malaysia; 3 Tropical Medicine Research Institute, Khartoum, Sudan; 4 Department of microbiology, faculty of medical laboratory sciences, National University, Khartoum, Sudan; 5 Centre for Chemical Biology, Universiti Sains Malaysia, 11900 Bayan Lepas, Penang Malaysia; 6 Department of Microbiology, Faculty of Medical Laboratory Science, International University of Africa, Khartoum, Sudan

**Keywords:** Methicillin -resistance *Staphylococcus aureus* (MRSA), Whole genome sequencing, Antibiotic resistant genes, Genome annotation, Sudan

## Abstract

**Background:**

Methicillin-resistant *Staphylococcus aureus* (MRSA) is known as a leading cause of morbidity and mortality. Investigation of the MRSA’s virulence and resistance mechanisms is a continuing concern toward controlling such burdens through using high throughput whole Genome Sequencing (WGS) and molecular diagnostic assays. The objective of the present study is to perform whole-genome sequencing of MRSA isolated from Sudan using Illumina Next Generation Sequencing (NGS) platform.

**Results:**

The genome of MRSA strain SO-1977 consists of 2,827,644 bp with 32.8% G + C, 59 RNAs and 2629 predicted coding sequences (CDSs). The genome has 26 systems, one of which is the major class in the disease virulence and defence. A total of 83 genes were annotated to virulence disease and defence category some of these genes coding as functional proteins. Based on genome analysis, it is speculated that the SO-1977 strain has resistant genes to Teicoplanin, Fluoroquinolones, Quinolone, Cephamycins, Tetracycline, Acriflavin and Carbapenems. The results revealed that the SO-1977, strain isolated from Sudan has a wide range of antibiotic resistance compared to related strains.

**Conclusion:**

The study reports for the first time the whole genome sequence of Sudan MRSA isolates. The release of the genome sequence of the strain SO-1977 will avail MRSA in public databases for further investigations on the evolution of resistant mechanism and dissemination of the -resistant genes of MRSA.

## Background

*Staphylococcus aureus* (*S. aureus*) is a human pathogen known to cause both nosocomial and community-acquired infections [1]. It has been identified, among other classes of bacteria, resistant against some antibiotics. One of the emerged resistant strain of *S. aureus* is Methicillin-resistant *Staphylococcus aureus* (MRSA) that is the leading cause of life-threatening infections even in countries with advanced health surveillance and maintenance systems [1, 2]. In Sudan, MRSA’s incidence rate has increased dramatically and has been reported to be associated with wound infection constituting substantial sources of the high morbidity and mortality rate [3]. Such emergence of resistant strains is due to the overuse of not developed antibiotics that ultimately makes real challenges at treatment. Therefore, there is an urgent need to uncover the genetic basis of their virulence and resistance mechanism for better understanding as well as addressing potential effective drug targets. Over the last decades, Whole-genome sequencing (WGS) technologies witnessed large volumes of produced data including mutant genes, cancer-causing genes and genes predisposing for certain diseases. Moreover, the advanced bench-top sequencers technique, applied in regular clinical laboratories [4] may result in enormous diagnostic developments and challenges [5]. Genomic materials of *S. aureus* strains have been studied to understand the mechanisms and virulence factors responsible for staphylococcal antibiotic resistance. The premier *S. aureus* genomes sequenced were; MRSA strains N315 and Mu50 [6] followed by other nine strains [7, 8]. The studies revealed that the length of staphylococcal genomes is about 2.8 Mbp with low GC content. The regions of staphylococcal genomes are well conserved, with many massive sequence blocks showing high variability [8]. Although a considerable number of the MRSA resistant to antimicrobials including Methicillin, Ofloxacin, Penicillin, Amikacin, and Vancomycin are reported in Sudan [9], the molecular investigations that help in understanding the mechanism of MRSA epidemics at the whole genome level are yet limited. The present study aims to analyse the whole genome sequence (WGS) of SO-1977 strain and subsequently evaluates the genomic diversity and genotypic prediction of the antimicrobial resistance of MRSA isolated from a patient in Sudan.

## Results

### Genome project history

The genome sequences of SO-1977 strain were deposited in GenBank® (WGS database). The result was summarized in (Table 1).

**Table 1:** Project Information

Property	SO-1977
Finishing quality	Complete
Libraries used	2 × 250 bp
Sequencing platforms	Illumina MiSeq
Fold coverage	122.26x
Assembly Method	SPAdes v. 3.9.0
GenBank ID	NFZY00000000
GenBank date of Release	27-JUL-2017
BIOPROJECT	PRJNA385553
BioSample	SAMN06894057
Locus Tag	CA803
Source Material Identifier	Wound
Project relevance	Medical

### Genomic features of strain SO-1977

As can seem from the data in Table 2, the draft genome sequence of *S. aureus* strain SO-1977 consisted of 2,827,644 bp with a 32.8% GC. The number of predicted coding sequences (CDS), tRNAs and rRNAs was 2629, 51 and 4 respectively. The final assembly contained 151 contigs with N50 of 62,783 bp length. The largest contig assembled was 146,886 bp length.

**Table 2:** Nucleotide and gene content’s levels of the MRSA SO-1977 genome

Attribute	Value
Genome size (bp)	2,827,644 bp
DNA G + C content	32.8%
Number of Contigs	151
N50	62,783 bp
rRNA genes	4
tRNA genes	51
ncRNAs	4
Protein-coding genes	2629
Pseudo Genes (total)	129
Pseudo Genes (frameshifted)	62
Pseudo Genes (incomplete)	32
Pseudo Genes (internal stop)	50
Pseudo Genes (multiple problems)	13
Genes assigned to SEED	1698

### Genome annotation using RAST (Fig. 1)

Whole-genome annotation of MRSA strain SO-1977 on RAST server revealed a total of 1970 genes belonging to 26 subsystems such as Cofactors, Vitamins, Prosthetic Groups, Pigments, Cell Wall and Capsule and Virulence, Disease and Defense. The graphical circular map of the SO-1977 genomes was shown in Fig. 2.

**Fig. 1:**
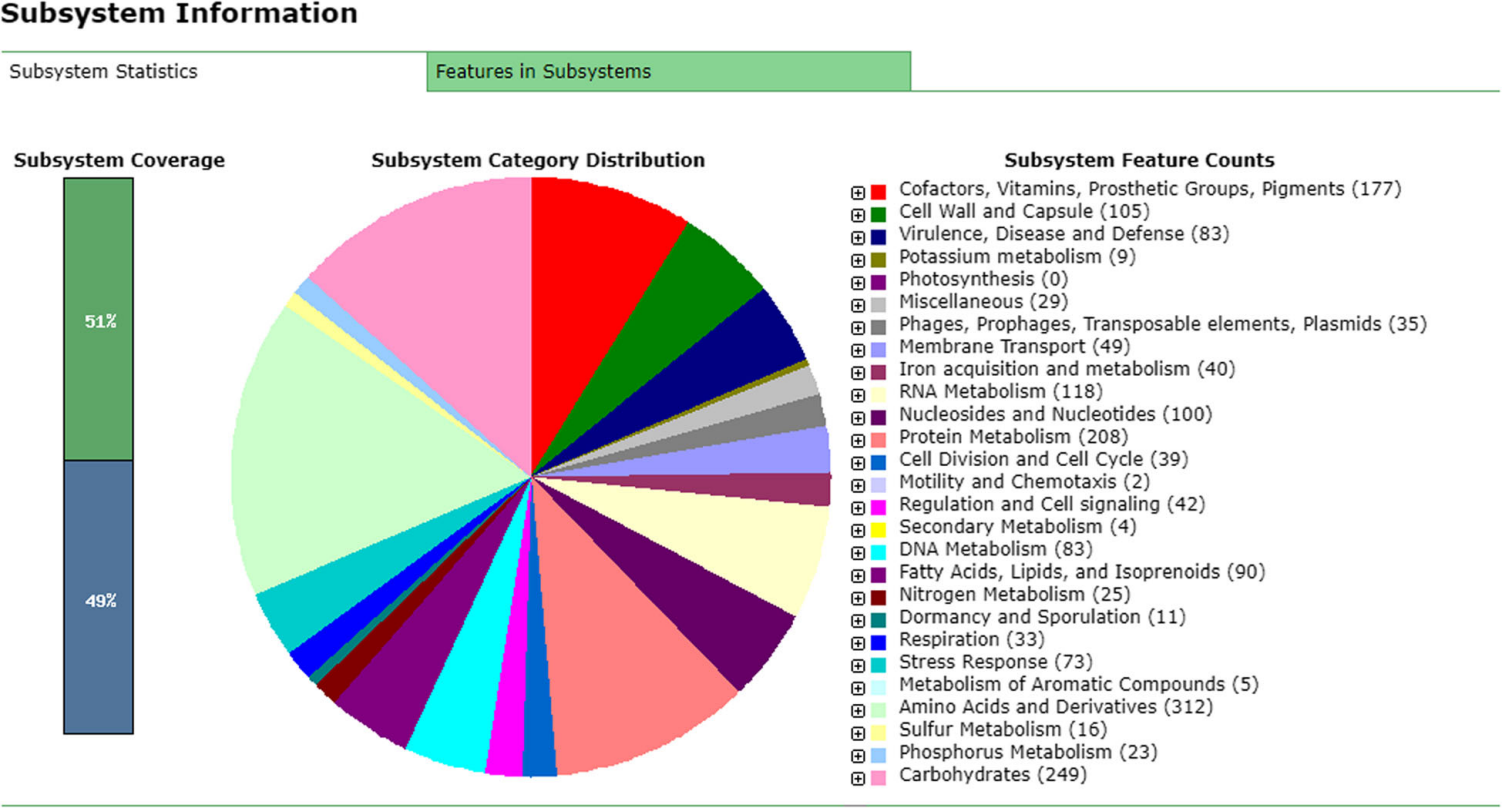
Summary of annotation for MRSA strain SO-1977 based on RAST subsystem

### Genes involved in virulence, disease and defence

Result revealed that 83 genes encoded for virulence, disease, and defence, 28 genes were annotated to be responsible for adhesion, 32 for antibiotic resistances and toxic compounds, 14 for Bacteriocins, ribosomally synthesized antibacterial peptides and 9 for invasion and intracellular resistance (Fig. 3). Some of these genes which coding functional proteins are Fibronectin binding protein, Chaperonin, Two-component response regulator BceR, Folylpolyglutamate synthase, Acetyl-coenzyme A, Carboxyl transferase beta chain, Colicin V production protein, MerR family, Multidrug resistance protein, Mercuric ion reductase and Arsenate reductase. The category of the cell wall and capsule system of peptidoglycan biosynthesis revealed that two genes have a relationship with conferring Methicillin resistance while one gene was related to Penicillin resistance.

**Fig. 2:**
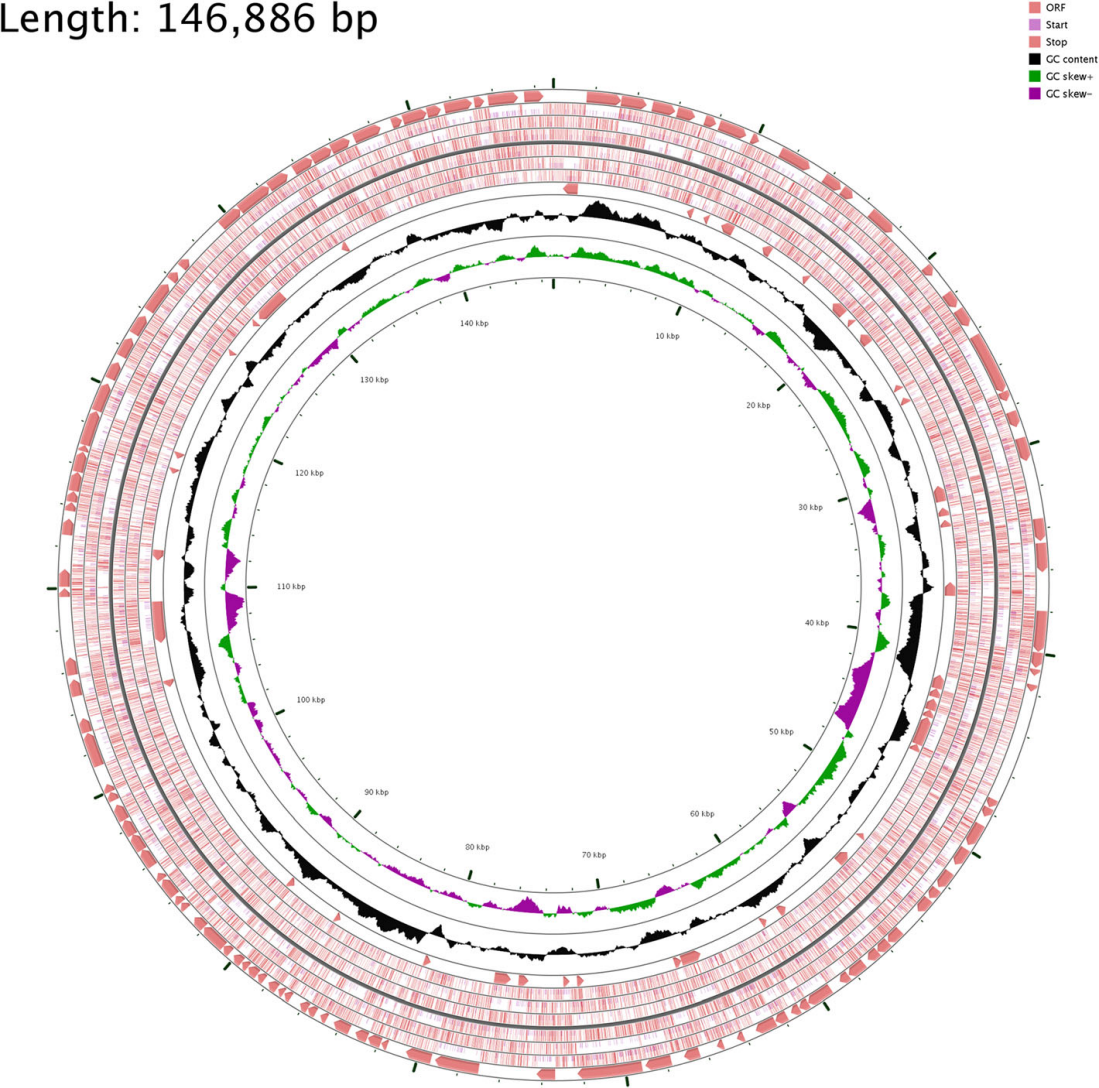
Circular map of the chromosome of the *S. aureus* SO − 1977. The innermost ring represents the SO − 1977 chromosome. The second ring (in black) plots the G + C content of the reference, followed by its G + C skew (in purple/green)

**Fig. 3:**
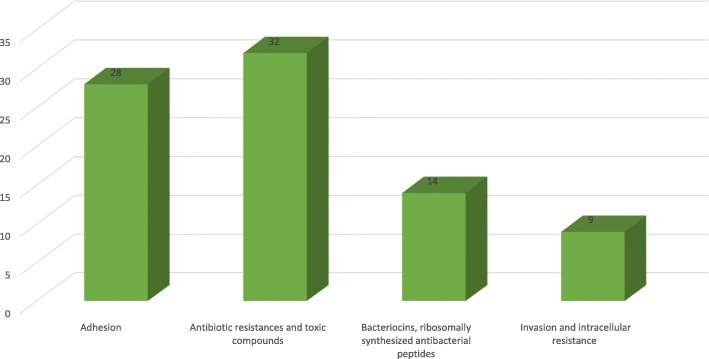
Bar diagram. Genes involved in category virulence disease and defense

### Phages, prophages, transposable elements, plasmids (Table 3)

The analysis revealed that 35 genes are encoding for Phages, Prophages, Transposable elements, Plasmid of which 33 were annotated to be responsible for Phages, Prophages and Pathogenicity islands.

**Table 3:** Systems included Phages, prophages, transposable elements, plasmids category

Category	Subcategory	Subsystem	Role
Phages, Prophages, Transposable elements, Plasmids	Phages, Prophages	Phage tail proteins	Phage tail protein/ Phage tail length tape-measure protein
		Phage replication	Single stranded DNA-binding protein/ Phage replication initiation protein/ DNA polymerase III alpha subunit/ DNA helicase, phage-associated
		Phage packaging machinery	Phage DNA packaging/ Phage terminase, small subunit/ Phage terminase, large subunit/ Phage portal protein
		Phage capsid proteins	Phage capsid and scaffold/ Phage head maturation protease/ Phage major capsid protein/ Phage capsid protein
		Phage lysis modules	Phage lysin, N-acetylmuramoyl-L-alanine amidase/ Phage holin
	Pathogenicity islands	Listeria Pathogenicity Island LIPI-1 extended	Phosphatidylinositol-specific phospholipase
			Zinc metalloproteinase precursor

### Resistant genes based comparative genomic analysis (Table 4)

The Genome annotation and comparison results by RSAT server have shown that SO-1977 strain possesses 29 genes that may be related to multi-drug resistance and the comparison between MRSA strains was shown that 23 resistant genes were present in all strains, two genes were only found in SO-1977 strain conferring resistance against Tetracycline. Furthermore, The SO-1977 strain was the only one having the norA gene providing resistance against Quinolone beside other six genes of the family MarR. Four genes that are responsible for anti- Methicillin resistance (LytH, MecI, Mec and MurE) were only found in MRSA252 strain. Also the results have shown that MRSA252 and MSSA476 are sharing a single common gene for anti-Methicillin resistance (HmrB).

**Table 5:** List of 16S rRNA

Strain	Accession no.	Seq match
*S. aureus*	L37597.1	1
*S. aureus MPU99*	AB353073.1	1
*S. aureus SA1*	AB305019.1	1
*S. aureus ATCC 14458*	DQ997837.1	1
*S. aureus subsp. anaerobius DSM 20714*	AY688031.1	1
*S. aureus K16–13*	AY859409.1	1
*S. aureus1*	AY144447.1	1

^a^Seq match score, the number of (unique) 7-base oligomers shared between your sequence and a given RDP sequence divided by the lowest number of unique oligos in either of the two sequences

### Phylogenetic analysis of nucleotide sequence of strain SO-1977

**Fig. 4:**
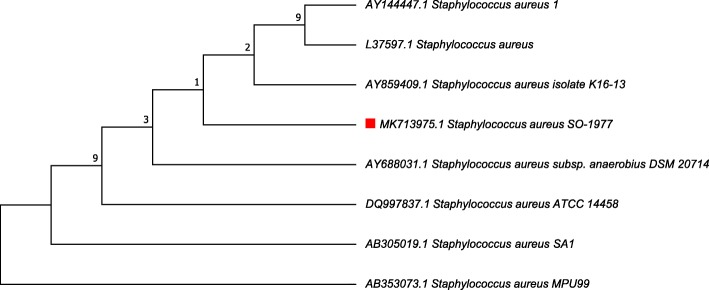
Neighbour-joining tree based on 16S rRNA gene sequences showing the Phylogenetic relationship between *Staphylococcus aureus* strain SO-1977 relative to other type strains within the *Staphylococcus aureus* in database. Bootstrap values (expressed as percentages of 100 replications) less than 50% are hidden

Result on the phylogenetic of 16S rRNA (MK713975) showed that the SO-1977 strain has the highest similarity with different *S. aureus* strain (Table 5) (Fig. 4).

**Table 4:** Summary of CDSs annotated to antibiotic resistance between SO-1977, MRSA252 and MSSA476 strains

No	SO-1977	MRSA252	MSSA476	Seed subsystem	Seed function	Length (bp)	Contig number
1	✓	✓		Peptidoglycan Biosynthesis	Methicillin resistance determinant MecA, transpeptidase	2007	Contig 000034
2	✓	✓	✓	Multidrug Resistance, 2-protein version Found in Gram-positive bacteria	Multidrug resistance protein [function not yet clear]	648	Contig 000037
3	✓	✓	✓	Multidrug Resistance, 2-protein version Found in Gram-positive bacteria	Membrane component of multidrug resistance system	1932	Contig 000037
4	✓	✓	✓	Multidrug Resistance, 2-protein version Found in Gram-positive bacteria	TetR family regulatory protein of MDR cluster	555	Contig 000037
5	✓			Tetracycline resistance, ribosome protection type	Tetracycline resistance protein TetM	1920	Contig 000042
6	✓			Tetracycline resistance, ribosome protection type	Translation elongation factor G	2082	Contig 000014
7	✓	✓	✓	Teicoplanin-resistance in Staphylococcus	Teicoplanin resistance associated membrane protein TcaA	1209	Contig 000002
8	✓	✓	✓	Teicoplanin-resistance in Staphylococcus	Teicoplanin resistance associated membrane protein TcaB	1383	Contig 000002
9	✓	✓	✓	Teicoplanin-resistance in Staphylococcus	Teicoplanin-resistance associated HTH-type transcriptional regulator TcaR	456	Contig 000002
10	✓			Quinolone resistance protein norA	none	1167	Contig 000007
11	✓	✓		Bicyclomycin resistance protein TcaB	none	1212	Contig 000002
12	✓	✓	✓	Transcriptional regulator, MarR family	None	456	Contig 000003
13	✓	✓	✓	Transcriptional regulator, MarR family	None	420	Contig 000005
14	✓	✓	✓	Transcriptional regulator, MarR family	None	210	Contig 000043
15	✓	✓	✓	Transcriptional regulator, MarR family	None	435	Contig 000030
16	✓	✓	✓	Transcriptional regulator, MarR family	None	441	Contig 000001
17	✓	✓	✓	Transcriptional regulator,MarR family	None	468	Contig 000030
18	✓	✓	✓	Resistance to fluoroquinolones	DNA gyrase subunit B	2403	Contig 000010
19	✓	✓	✓	Resistance to fluoroquinolones	DNA gyrase subunit A	2661	Contig 000011
20	✓	✓	✓	Resistance to fluoroquinolones	Topoisomerase IV subunit B	1998	Contig 000010
21	✓	✓	✓	Resistance to fluoroquinolones	Topoisomerase IV subunit A	2403	Contig 000010
22	✓	✓	✓	Beta-lactamase	Beta-lactamase	1500	Contig 000002
23	✓	✓	✓	Beta-lactamase	Beta-lactamase repressor BlaI	381	Contig 000067
24	✓	✓	✓	Multidrug Resistance Efflux Pumps	Acriflavin resistance protein	3168	Contig 000001
25	✓	✓	✓	Multi antimicrobial extrusion protein (Na(+)/drug antiporter), MATE family of MDR efflux pumps	Multidrug Resistance Efflux Pumps	1356	Contig 000005
26		✓	✓	Methicillin resistance in staphylococci	HmrB protein involved in methicillin resistance	1600	NC_002952
27	✓	✓	✓	Methicillin resistance in staphylococci	Beta-lactamase repressor BlaI	381	Contig 000067
28	✓	✓	✓	Methicillin resistance in staphylococci	FmhA protein of FemAB family	1251	Contig 000002
29		✓		Methicillin resistance in staphylococci	LytH protein involved in methicillin resistance	1600	NC_002952
30	✓	✓		Methicillin resistance in staphylococci	Methicillin resistance regulatory sensor-transducer MecR1	1600	Contig 000034/ NC_002952
31		✓		Methicillin resistance in staphylococci	Methicillin resistance repressor MecI	1600	NC_002952
32		✓		Methicillin resistance in staphylococci	Transposase for insertion sequence-like element IS431mec	1296	NC_002952
33		✓		Methicillin resistance in staphylococci	UDP-N-acetylmuramoylalanyl-D-glutamate--2,6-diaminopimelate ligase	1600	NC_002952
34	✓	✓	✓	Methicillin resistance in staphylococci	Beta-lactamase regulatory sensor-transducer BlaR1	1089	Contig 000067

## Discussion

The present study reported the first genome sequence of *S. aureus* (MRSA) isolated from Sudan to have phylogenetic allocation using the 16S rRNA gene to represent the evolutionary relationships of the bacteria. In this study, the phylogenetic analysis of the complete 16S rRNA gene sequence of strain SO-1977 (MK713975) has shown that the strain should be assigned to the genus *Staphylococcus*. The annotated draft genome sequence of SO-1977 strain was 2827,644 bp length containing 2629 coding sequences (CDS). Moreover, the WGS data was used to investigate antimicrobial resistance and virulence mechanism. The multi-drug resistance of this isolate might be generated by the ability of these bacteria to accumulate multiple genes on the resistance (R) plasmids coding for a single drug resistance within a single cell or by the increased expression of genes that code for multi-drug efflux pumps, extruding a wide range of drugs [10]. In this study, *S. aureus* (MRSA) isolated from Sudan has been demonstrated to possess different resistance mechanisms which can be attributed to the use of resistant genes TcaR, TcaA, TcaB, TetR, TetM, PBP2a (MecA), or by secretion of enzymes (DNA gyrase subunit A, DNA gyrase subunit B, Topoisomerase IV subunit A, Topoisomerase IV subunit B and Beta-lactamase repressor) allowing it to use the efflux pump mechanism. In addition, six putative MarR family transcriptional regulators in the SO-1977 genome were identified. These were recognised as a widely conserved group of multiple antibiotic resistance regulators that respond to a wide range of antibiotics [11]. The MRSA characteristic phenotype is due to the presence of mecA, which encodes a penicillin-binding protein (PBP), PBP2a, with reduced affinity for b-lactams. MecA is embedded in a large heterologous chromosomal cassette, the SCCmec element. Some MRSA strains carry upstream to the mecA gene such as the regulatory genes mecI-mecR1 that encoding for a repressor and a sensor/inducer of the mecA expression, respectively [12]. In this study, MecA and MecR1 were found in SO-1977 and MRSA252, while the mecI was found only in MRSA252. This result suggested that the existence of yet unidentified additional determinants involved in the transcriptional control of mecA gene and point to a revision of the mecA regulatory mechanism in MRSA SO-1977 strain. The antibiotic sensitivity tests demonstrated that the isolate is resistant to discsoxacillin and cefoxitin. The result of the WGS confirmed the resistance of the isolate to the antibiotics and expanded it to include Teicoplanin, Fluoroquinolones, Quinolone, Cephamycins, Tetracycline, Acriflavin and Carbapenems. Such a result should be considered while planning an effective treatment protocol. The antibiotic-resistant genes of SO-1977, MRSA252 and MSSA476 revealed that the SO-1977 strain isolated from Sudan is complicated and has a wide range of cross-antibiotic resistance.

## Conclusion

The current study is the first of its kind in Sudan to give an insight of an important antibiotic resistant bacterial strain, MRSA SO-1977. The SO-1977 strain is resistant to Teicoplanin, Fluoroquinolones, Quinolone, Cephamycins, Tetracycline, Acriflavin and Carbapenems. This study strongly suggests that other yet unidentified determinants are involved directly or indirectly in the transcriptional control of the mecA gene in SO-1977 strain. The SO-1977 strain has a wide range of antibiotic resistance compared to other strains. The whole genome of SO-1977 strain can provide a genetic background of virulence, antibiotic resistance and Phages of the MRSA species in Sudan.

## Methods

### Sample preparation

A wound swab specimen was collected from a patient at Soba Hospital, Khartoum, and was inoculated in sheep blood agar and mannitol salt agar at 37 °C for 24 h. For the purpose of colonies identification, standard procedures and tests were performed including gram stain, catalase, coagulase, and DNase tests were used to identify the colonies [13]. The positive cultures for *S. aureus* were then suspended with a concentration similar to turbidity standard equivalent to 0.5% McFarland and streaked on Mueller-Hinton agar (MHA). Oxacillin (6μg\ml) and cefoxitin (30μg\ml) antimicrobial disc were positioned at suitable distances on the bacterial lawns on MHA at 33 °C for 24 h. The antibiotic resistance profiling of the strain against a broader range of antibiotics was not performed as a limitation of the study. The growth inhibition zones were then measured according to the standard Kirby –Bauer disc diffusion method and NCCLs guidelines using a calliper [14]. In which the revealed measurements were indicatives of resistant colonies of MRSA strain.

### Genomic DNA extraction and sequencing library preparation

Bacterial DNA was extracted using Qiagen Kit following the manufacturer instructions. The concentration and purity of the resultant DNA were photo-metrically determined using a Nano-drop (Thermofisher®). About 5 μg of genomic DNA (A260/280 = 1.93) was used for library preparation and 4 nm of genomic DNA was used as an input for the Nextera XT kit (Illumina). Then samples were targeted for bar-coding using forward (N702) and reverse (N702) primers in 12 cycles of amplification in the PCR machine. Libraries were then quantified on the Bioanalyzer (Agilent Technologies) and combined with an equimolar mixture. Finally, 0.19 ng/ ml was used as an input for Next-generation sequencing (NGS) and libraries were sequenced on a single run on the Illumina MiSeq instrument (250 bp paired-end reads).

### Bacterial genome sequencing and assembly

Poor-quality and adaptor-containing reads were filtered and trimmed using BBTools version 36 [15]. Good quality sequencing reads were assembled using SPAdes version 3.5.0. For the prediction of tRNA and rRNA genes, ARAGORN 1.2.34 and RNAmmer1.2 were used, respectively [16, 17]. The protein-coding genes were then predicted using Prodigal 2.60 [18] as well as their function by using BLASTN 2.2.25+ [19] and followed by detecting sequence homologs through searching for various sequence domain databases using HMMER 3.0 (http://hmmer.org/).

### Genome annotation

The final draft genome sequence of *S. aureus* SO-1977 was used for annotation using RAST [20] and NCBI Prokaryotic Genome Annotation Pipeline [21]. The annotated genes were exported from the RAST server into an excel table and manually compared for genomic features. The antibiotic resistance genes of the *S. aureus* SO-1977, *S. aureus* MRSA252 (PRJNA265) and *S. aureus* MSSA476 (PRJNA116329) were retrieved from RAST server then the comparison was done [22]. The graphical circular map of the genomes was made by CGView server [23].

### Phylogenetic analysis of strain SO-1977 using 16S rRNA

The 16S rRNA sequences were edited and assembled to a final length of 1545 bp, GenBank accession number (MK713975). All reference sequences of the 16S rRNA genes of *S.aureus* used in this study were obtained from GenBank® (https://www.ncbi.nlm.nih.gov/genbank/) using the RDP 11.5, Seqmatch: version 3 (https://rdp.cme.msu.edu/). DNA sequence alignment was performed using MUSCLE v. 3.8.31 (http://www.ebi.ac.uk/Tools/msa/muscle/) on the European Bioinformatics Institute (EBI) homepage. Once the alignment was completed, the phylogenetic tree was drowning as the evolutionary distances were computed using the Maximum Likelihood method implemented in MEGA6 version 6 [24] in all positions containing gaps and missing data were eliminated.

### Sequence data access

The genomic data of this study were deposited publicly in DDBJ/ENA/GenBank® under Accession: NFZY00000000, BioProject: PRJNA385553 and Biosample: SAMN 06894057.

## Abbreviations

CDS: Coding sequences
MRSA: Multidrug-resistant *Staphylococcus aureus*
NGS: Next-generation sequencing
WGS: Whole genome sequencing
RAST: Rapid Annotation using Subsystem Technology
